# Synopsis of the African species of *Acropimpla* Townes, 1960 (Hymenoptera, Ichneumonidae), with new faunistic records and descriptions of males

**DOI:** 10.3897/BDJ.14.e215045

**Published:** 2026-10-07

**Authors:** Emil M. Österman, Tapani Hopkins, Kari M. Kaunisto, Yves Braet, Ilari E. Sääksjärvi

**Affiliations:** 1 Department of Biodiversity Sciences, University of Turku, Turku, Finland Department of Biodiversity Sciences, University of Turku Turku Finland; 2 Institut Royal des Sciences Naturelles de Belgique, Brussels, Belgium Institut Royal des Sciences Naturelles de Belgique Brussels Belgium

**Keywords:** biodiversity, Côte d'Ivoire, distribution, Malaise trap, morphological variation, Pimplinae, taxonomy, Uganda

## Abstract

**Background:**

*Acropimpla* is a nearly cosmopolitan genus of Darwin wasps (Hymenoptera, Ichneumonidae, Pimplinae), with three known species from Africa. However, all the current African records are from east Africa and no males are known. We report the specimens we collected by Malaise trapping in Uganda and Côte d'Ivoire and summarise the known information about the African species.

**New information:**

We describe the males of *A. alboscutellaris* (Szépligeti, 1908) and *A. lutea* Varga, 2026, which resemble the females, but are smaller and have darker metasomal tergites. Furthermore, the male of *A. alboscutellaris* has an unintercepted nervellus, which is intercepted in the female. We present two new country-level records of *A. alboscutellaris* from Uganda and Côte d'Ivoire, the latter of which constitutes the first genus-level record in west Africa. The Ugandan data on *A. lutea* suggests that it is a dry-season-dominant forest species.

## Introduction

*Acropimpla* Townes, 1960 is a moderately large genus within the tribe Ephialtini of the subfamily Pimplinae of Darwin wasps (Hymenoptera, Ichneumonidae) which includes 48 described species (Yu et al. 2016, Khalaim and Lopez-Ortega 2022, Khalaim and Ruíz-Cancino 2022, Varga 2026). The genus has a nearly cosmopolitan distribution (Yu et al. 2016), being, as far as is known, absent only from the Antarctic and Neotropical Regions and it is most species-rich in the Oriental Region (Baltazar 1966, Gupta and Tikar 1976, Liu et al. 2010, Varga and Reshchikov 2015). Species of *Acropimpla* are known as idiobiont parasitoids mainly of immature Lepidoptera, especially Tortricidae and Pyralidae (Yu et al. 2016).

Three species of *Acropimpla* are known from Africa (Szépligeti 1908, Seyrig 1932, Varga 2026), for which no males are known and which lack distributional records outside east Africa and Madagascar. Interestingly, all of these species have an open areolet (i.e. lack the fore-wing vein 3rs-m), which is highly rare in their non-African congeners, suggesting the African species may have inherited the open areolet from their last common ancestor.

In this article, we summarise known information about the African species of *Acropimpla* and, based on an extensive one-year sampling in Uganda (reported by Hopkins et al. (2019a), Hopkins et al. (2019b), Österman et al. (2026a)) and samples from Côte d'Ivoire, present as new information the descriptions of the males of two species, two country-level records of one species and ecological data of one species.

## Materials and methods

We collected the mainland specimens studied here during two sampling campaigns: an extensive campaign in Uganda (east Africa) and a smaller in Côte d'Ivoire (west Africa). The Ugandan material includes samples collected with 34 Malaise traps that operated for one year (2014–2015) in Kibale National Park. The traps were placed in habitats that varied across a successional gradient from primary forest and swampy primary forest, through disturbed forest and clearcut former plantation, to farmland. The sampling effort totalled 382.4 trap months, of which 22.6 trap months were damaged samples or otherwise provided, at most, a partial catch of ichneumonids. The study area and sampling campaign are described in greater detail by Hopkins et al. (2019a) and Hopkins et al. 2019b. The material from Côte d'Ivoire includes samples collected with one Malaise trap running for one and a half years (April 2018–November 2019) in highland forest on Mont Tonkoui (18.7 trap months). For the Malagasy material included here, we examined only images of the holotype.

We photographed the specimens using a Sony Alpha 7R V camera body mounted on a macro rail, which enabled us to control and incrementally move the camera between shots. Photos were captured using an extension tube, a relay lens and Mitutoyo Plan Apo objectives with 2.5× and 5× magnifications. We captured multiple images at successive focal depths and combined them using the software Helicon Focus v. 7.7.5 (by Helicon Soft Ltd) to produce composite layer images with extended depth of field. We carried out final image adjustments in Adobe Photoshop CC to ensure accurate representation of the specimen's morphological features. Morphological terminology follows Broad et al. (2018) and the taxonomic history is derived from Townes and Townes (1973).

### Repositories

**MNHN** Muséum national d'Histoire naturelle, Paris, France (Antoine Guiguet).

**ZMUT** Zoological Museum, University of Turku, Turku, Finland (Ilari Sääksjärvi).

## Data resources

The complete data of the 56 examined specimens from Uganda and one examined specimen from Côte d'Ivoire is in the Zenodo repository (Österman et al. 2026b). The repository also has the basic (summary) data of the examined specimens, an image of the hind-wing of our male *Acropimpla alboscutellaris* (ZMUT.11680) and copies of the images of *Acropimpla* in this article (for ease of download).

## Taxon treatments

### Acropimpla

Townes, 1960

6369374F-6C32-5A08-A149-EB2D7672E36B

Selenaspis Roman, 1910: 191 (homonym of *Selenaspis* Bleeker, 1858 - Chordata, Ariidae)
Type: *Hemipimpla alboscutellaris* Szépligeti, 1908 (orig. des.)
Acropimpla Townes, 1960: 159 (nomen novum for *Selenaspis* Roman, 1910)
Type: *Charitopimpla leucostoma* Cameron, 1907 (orig. des.)

#### Diagnosis

Clypeus more or less flat, apically notched; mandible usually with light-coloured area; eyes not strongly notched opposite antennal sockets; occipital carina medially dipped or weakly arched downwards, sometimes absent at mid-line; propodeum short and arched; fore-wing vein 3rs-m present or absent, when present, areolet receiving 2m-cu at or slightly basad of its distal corner; nervellus intercepted below middle (but rarely at middle or unintercepted); first metasomal tergite short and wide; second tergite with oblique grooves cutting off basolateral corners; ovipositor laterally compressed, dorsal valve concave (but rarely straight) distad of nodus, apex of ventral valve with oblique teeth (diagnosis adapted from Pham et al. (2011) and Varga (2026)).

### Acropimpla alboscutellaris

(Szépligeti, 1908)

1BB17442-5077-53DB-8034-11385FC1BB43

Hemipimpla alboscutellaris Szépligeti, 1908: 83 (orig. des.)
Selenaspis alboscutellaris ; Roman 1910: 191 (comb. nov.)
*Hemipimpla*? *alboscutellaris*; Schulz 1911: 28 (incertae sedis)
*Hemipimpla*? *alboscutellaris*; Morley 1914: 108 (incertae sedis)
Acropimpla alboscutellaris ; Townes & Townes 1960: 159 (comb. nov.)

#### Materials

- **Type status:** Other material. **Occurrence:** recordedBy: Y. Braet & A. Gué; individualCount: 1 ; sex: female; occurrenceID: 0B2125AF-60F8-553F-B546-CE3AD732AB17; **Location:** country: Côte d’Ivoire; locality: Mont Tonkoui; **Event:** samplingProtocol: Malaise trap; eventDate: 30 Sep-7 Oct 2018; habitat: highland forest; **Record Level:** institutionCode: ZMUT; **Material Entity:** digitalSpecimenID: ZMUT.49881
- **Type status:** Other material. **Occurrence:** recordedBy: T. Hopkins; individualCount: 2; sex: 1 female, 1 male; occurrenceID: 8A4032F3-D586-5D0C-8988-16506B05C0E2; **Location:** country: Uganda; locality: Kibale National Park, Kanyawara; **Event:** samplingProtocol: Malaise trap; eventDate: 29 Jan–13 Feb 2015; habitat: forest; **Record Level:** institutionCode: ZMUT; **Material Entity:** digitalSpecimenID: ZMUT.3153, ZMUT.11680

#### Description

**Figure 1.**
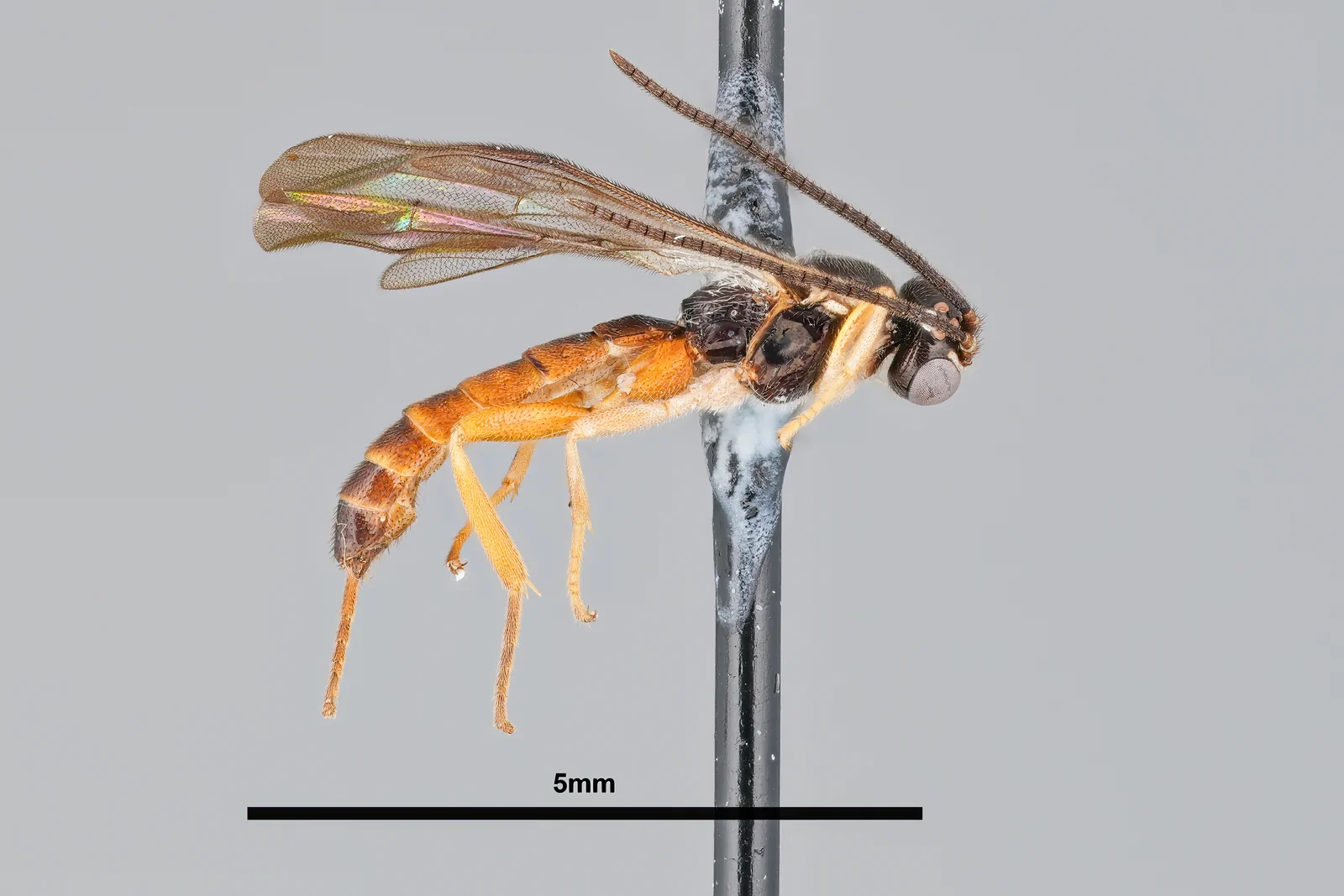
*Acropimpla alboscutellaris*, male from Uganda (ZMUT.11680), habitus in lateral view.

**Figure 2.**
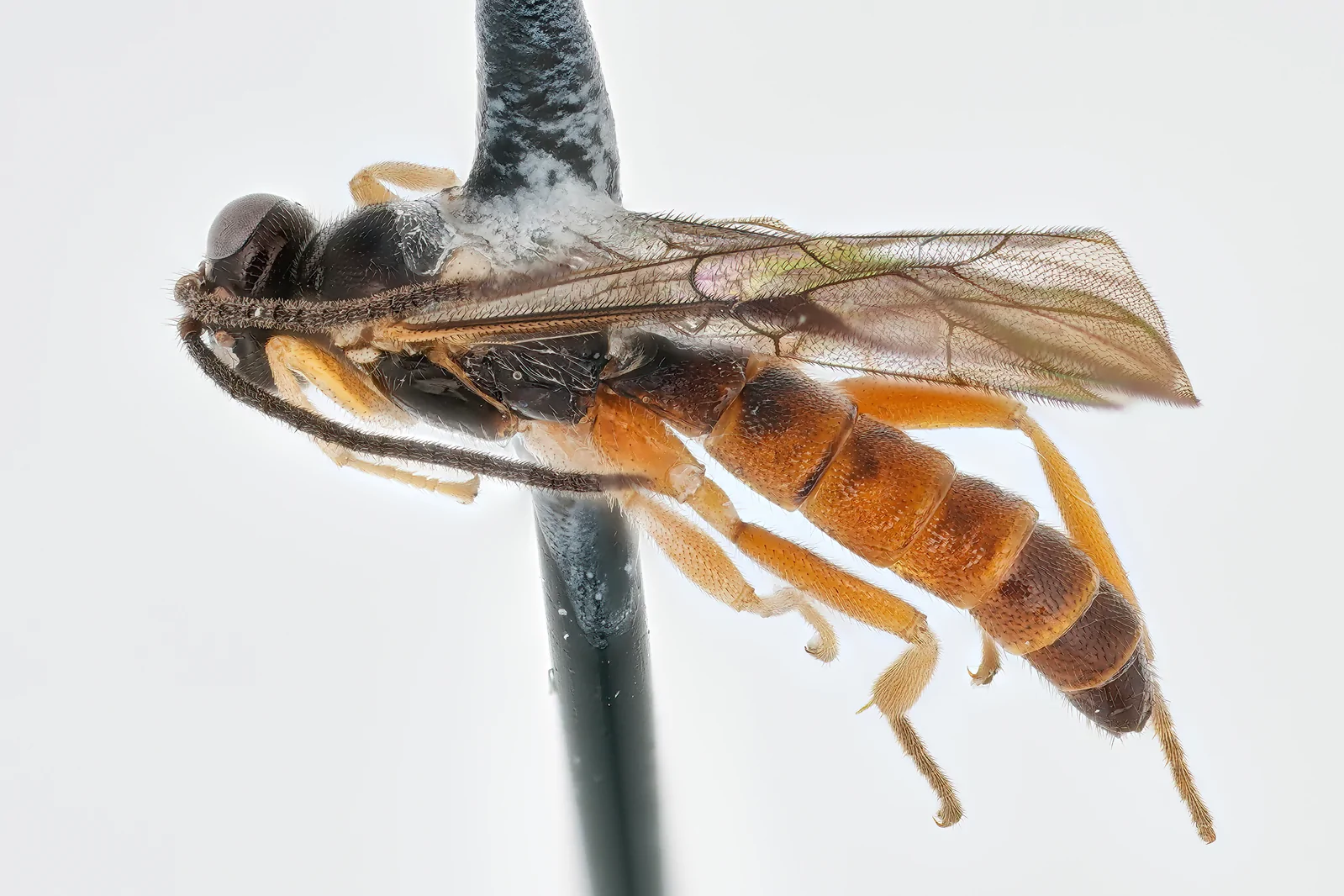
*Acropimpla alboscutellaris*, male from Uganda (ZMUT.11680), habitus in dorsolateral view.

**Figure 3.**
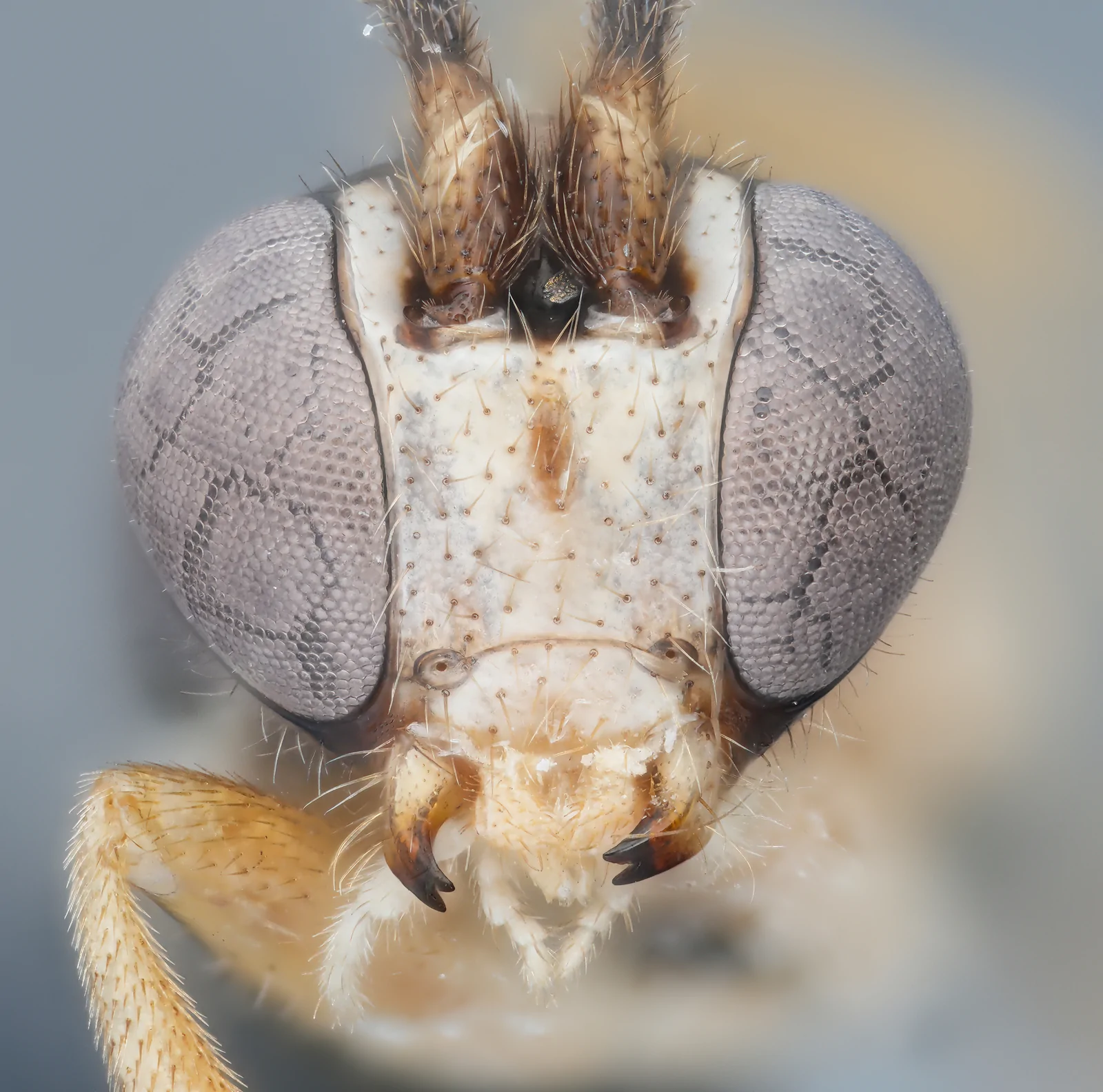
*Acropimpla alboscutellaris*, male from Uganda (ZMUT.11680), face in frontal view.

**Figure 4.**
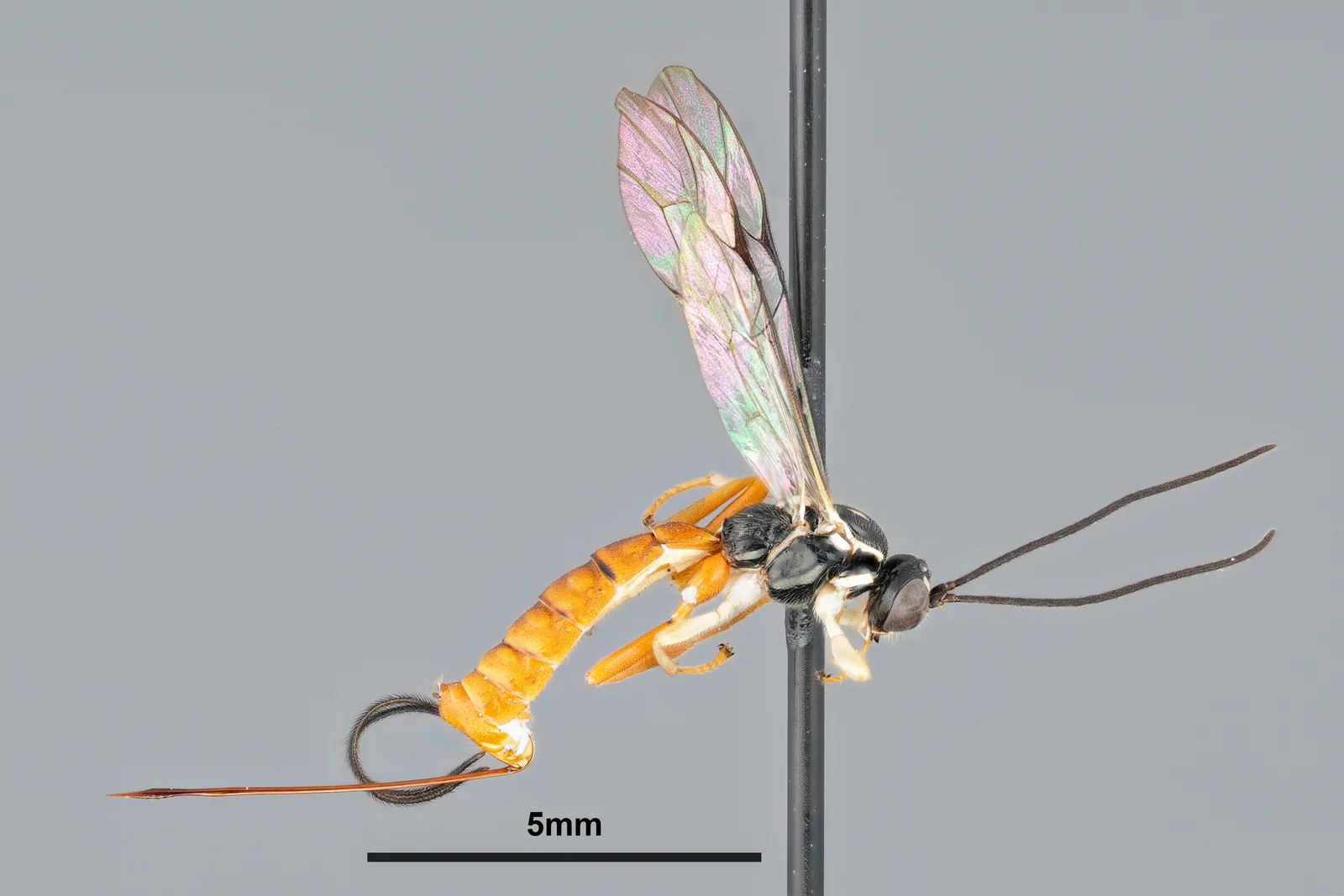
*Acropimpla alboscutellaris*, female from Côte d'Ivoire (ZMUT.49881), habitus in lateral view.

The male (Figs 1, 2, 3) is similar to the female (Fig. 4), except for the following: antenna with 18 flagellomeres (20–22 in females); nervellus unintercepted (detailed image in Österman et al. (2026b)); intercepted below the middle in female); face with faint vertical black stripe (stronger in female); metasomal tergites with extensive black areas, especially on basal and apical tergites (fully orange in female, except for occasional black bands along the apical margins). The male is also significantly smaller: fore-wing length about 4.4 mm vs. 6.0 mm in examined females.

#### Diagnosis

This species can be separated from the other known African species of the genus by any of the following: nervellus intercepted below middle in female; mesosoma (excluding legs) black with white markings.

#### Distribution

Kenya (Varga 2026); Tanzania (Szépligeti 1908). **New**: Côte d'Ivoire; Uganda.

#### Biology

No host records are known.

#### Notes

The female from Côte d’Ivoire shows minimal variation compared to the Ugandan female and the holotype, but it differs in having a black band along the apical margin of tergites 2–4 (very faint on tergites 3 and 4). Variation in the occurrence of apical black bands on the tergites and black areas on male tergites is common in Pimplinae (see, for example, Österman et al. (2026c)). The intraspecific difference (between males and females) in the interception of the nervellus is not common in our experience. This may occur because males, due to their often smaller size, usually have weaker characters than females and the females have the distal abscissa of CU quite thin. However, the male of *A. lutea* has an intercepted nervellus despite the similarly weak distal abscissa of CU in the female. Hence, it is possible that larger male specimens have an intercepted nervellus, but this remains to be confirmed.

### Acropimpla lucifugus

(Seyrig, 1932)

66AAADF3-7413-5213-8CA2-4F9FF0162B6A

Selenaspis lucifugus Seyrig, 1932: 38 (orig. des.)
Acropimpla lucifuga ; Townes & Townes 1960: 160 (comb. nov.)

#### Materials

- **Type status:** Holotype. **Occurrence:** recordedBy: A. Seyrig; individualCount: 1; sex: female; occurrenceID: 1619695D-49A4-548E-A03C-478B4AD39BC4; **Location:** country: Madagascar; locality: Vatondrangy; **Event:** eventTime: Dec 1929; habitat: mountain forest; **Record Level:** institutionCode: MNHN; **Material Entity:** digitalSpecimenID: EY9433

#### Diagnosis

**Figure 5.**
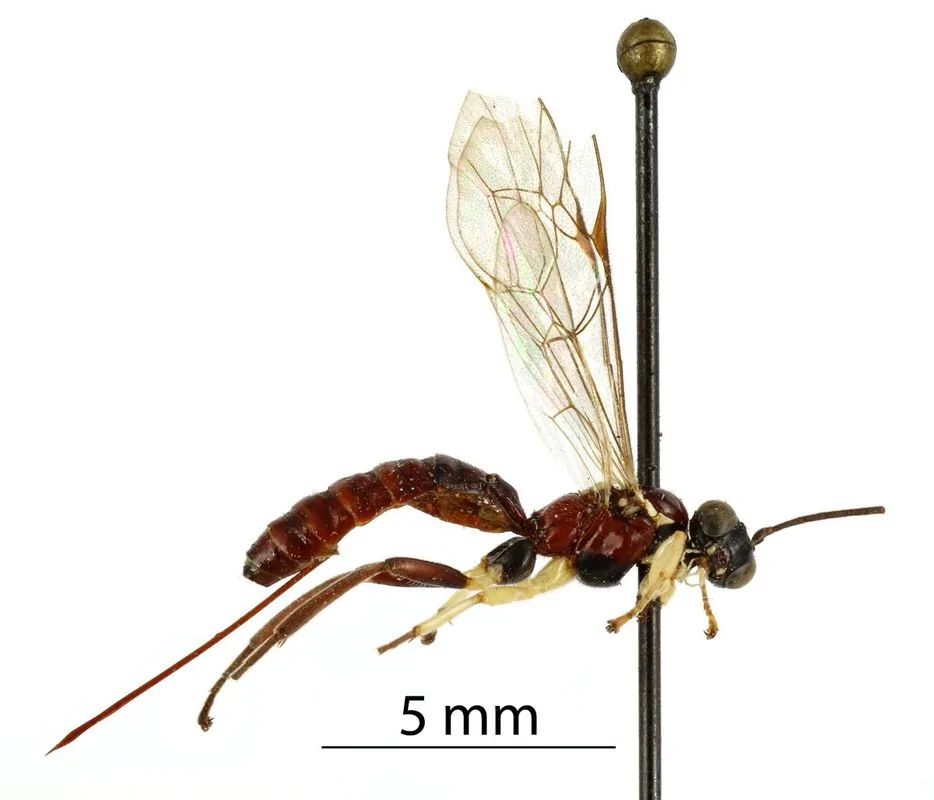
*Acropimpla lucifugus*, holotype female (EY9433), habitus in lateral view. Credit: RECOLNAT (ANR-11-INBS-0004) - Christophe Hervé - 2014. © Muséum national d'Histoire naturelle, Paris.

**Figure 6.**
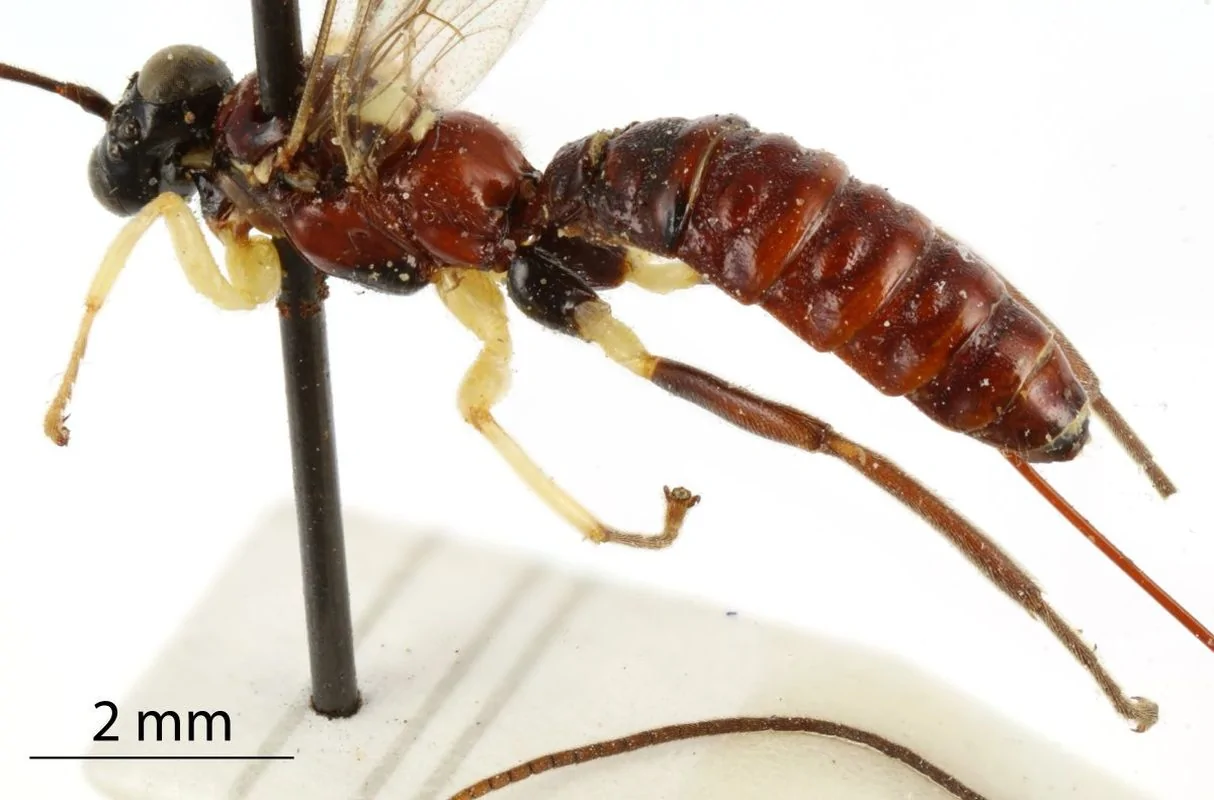
*Acropimpla lucifugus*, holotype female (EY9433), habitus in dorsolateral view. Credit: RECOLNAT (ANR-11-INBS-0004) - Christophe Hervé - 2014. © Muséum national d'Histoire naturelle, Paris.

**Figure 7.**
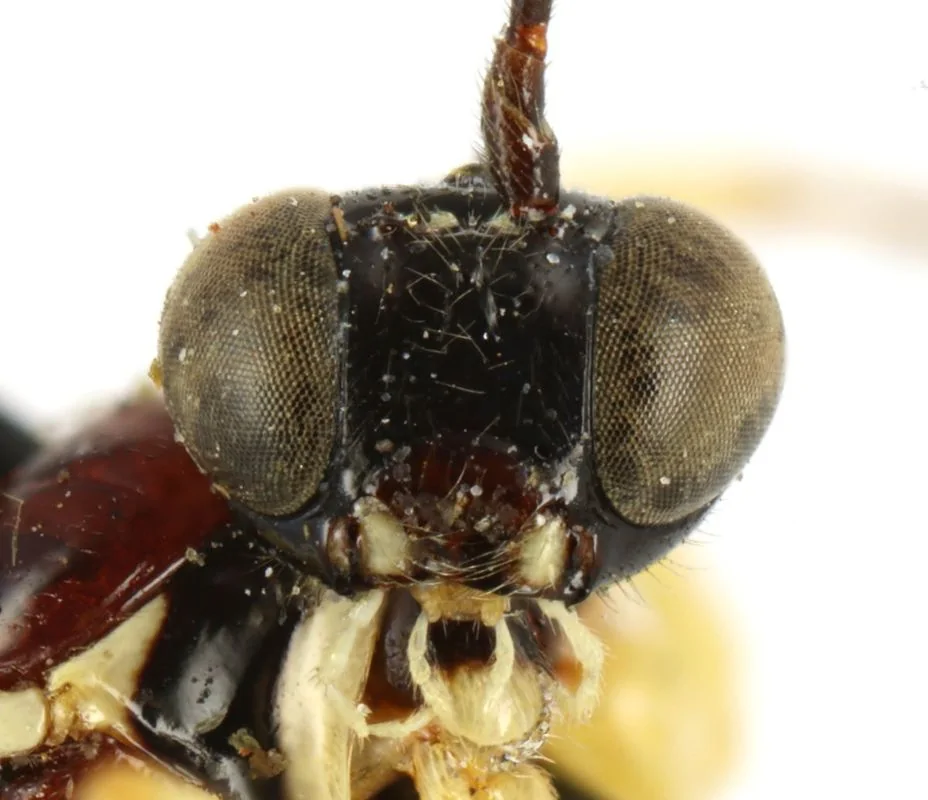
*Acropimpla lucifugus*, holotype female (EY9433), face in frontal view. Credit: RECOLNAT (ANR-11-INBS-0004) - Christophe Hervé - 2014. © Muséum national d'Histoire naturelle, Paris.

This species (Figs 5, 6, 7) can be separated from the other known African species of the genus by any of the following: nervellus unintercepted in female; face entirely black; mesosoma (excluding legs) burgundy red with black and white markings.

#### Distribution

Madagascar (Seyrig 1932).

#### Biology

No host records are known.

#### Notes

This is, to our knowledge, the only species of the genus whose female has an unintercepted nervellus. The male is not known, but should also have the same character state.

### Acropimpla lutea

Varga, 2026

A5210E88-D9CA-5181-9511-39465A58054E

#### Materials

- **Type status:** Other material. **Occurrence:** recordedBy: T. Hopkins; individualCount: 56; sex: 54 females, 1 male, 1 unknown; occurrenceID: 6D057F12-13DB-510E-81E0-E671704BCE95; **Location:** county: Uganda; locality: Kibale National Park, Kanyawara; **Event:** samplingProtocol: Malaise trap; eventDate: 9 Sep 2014-26 Aug 2015; habitat: forest; **Record Level:** institutionCode: ZMUT; **Material Entity:** digitalSpecimenID: ZMUT.1620, ZMUT.2110, ZMUT.2278, ZMUT.2831, ZMUT.2846, ZMUT.2902, ZMUT.3390, ZMUT.3426, ZMUT.3440, ZMUT.3588, ZMUT.4309, ZMUT.4330, ZMUT.5337, ZMUT.5341, ZMUT.6026, ZMUT.6028, ZMUT.8954, ZMUT.10768, ZMUT.10772, ZMUT.11914, ZMUT.12029, ZMUT.14978, ZMUT.15840, ZMUT.16247, ZMUT.16797, ZMUT.16949, ZMUT.17516, ZMUT.17673, ZMUT.18000, ZMUT.18052, ZMUT.18074, ZMUT.18236, ZMUT.19396, ZMUT.19872, ZMUT.19972, ZMUT.20273, ZMUT.20365, ZMUT.20376, ZMUT.20377, ZMUT.20792, ZMUT.20805, ZMUT.20876, ZMUT.25117, ZMUT.25649, ZMUT.25669, ZMUT.25805, ZMUT.26173, ZMUT.26712, ZMUT.26787, ZMUT.26928, ZMUT.27233, ZMUT.27653, ZMUT.28386, ZMUT.28565, ZMUT.28655, ZMUT.29054

#### Description

**Figure 8.**
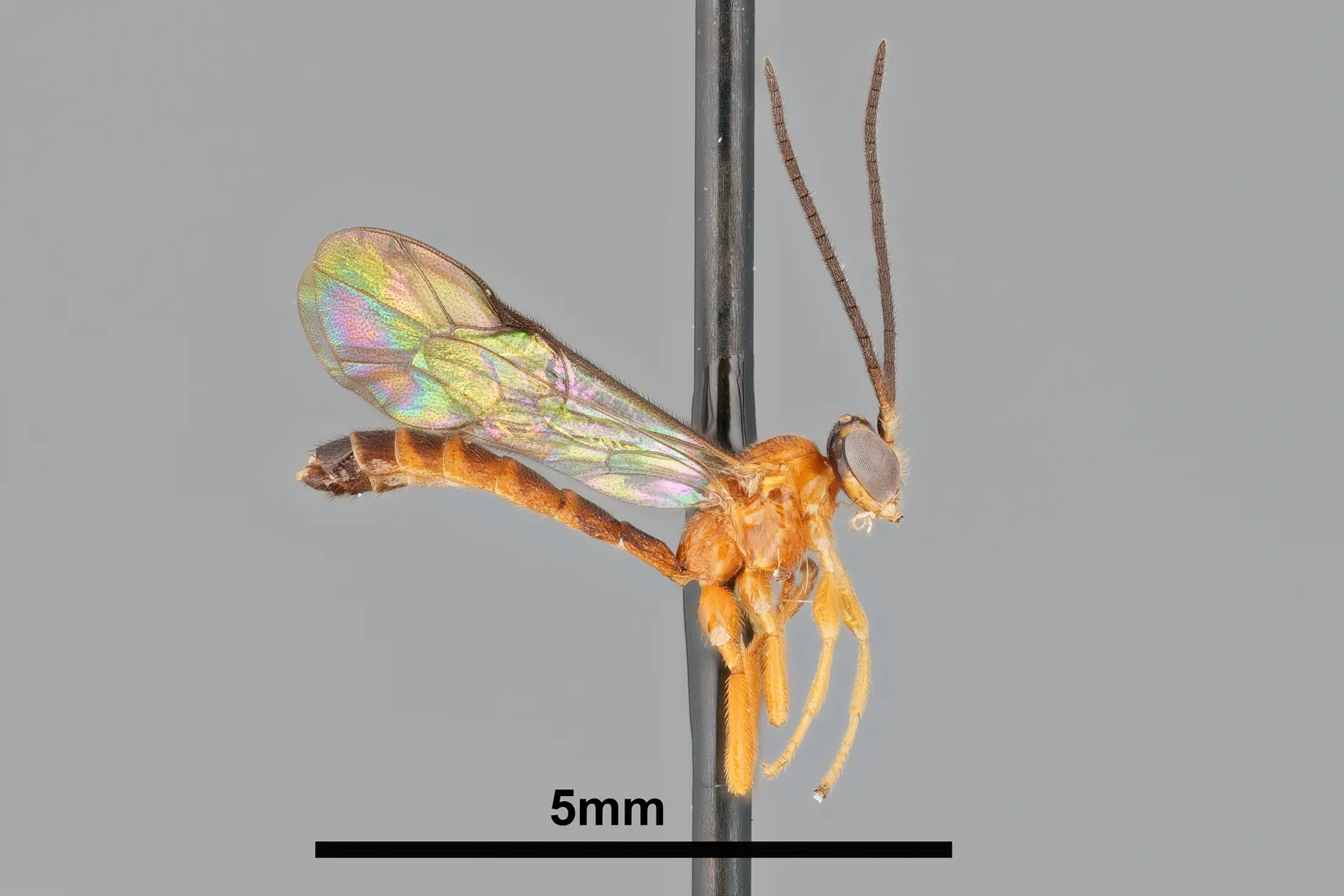
*Acropimpla lutea*, male from Uganda (ZMUT.2846), habitus in lateral view.

**Figure 9.**
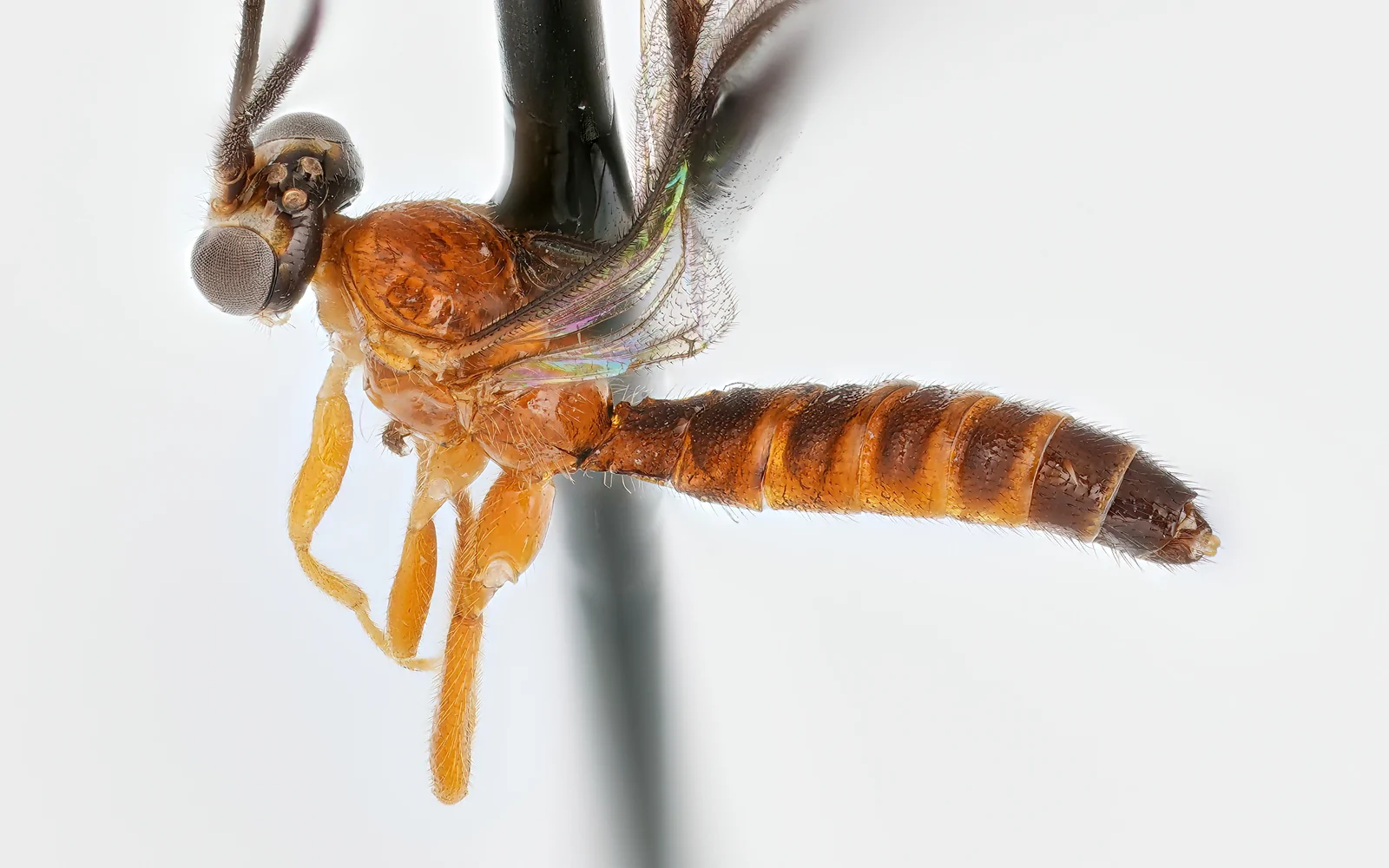
*Acropimpla lutea*, male from Uganda (ZMUT.2846), habitus in dorsolateral view.

**Figure 10.**
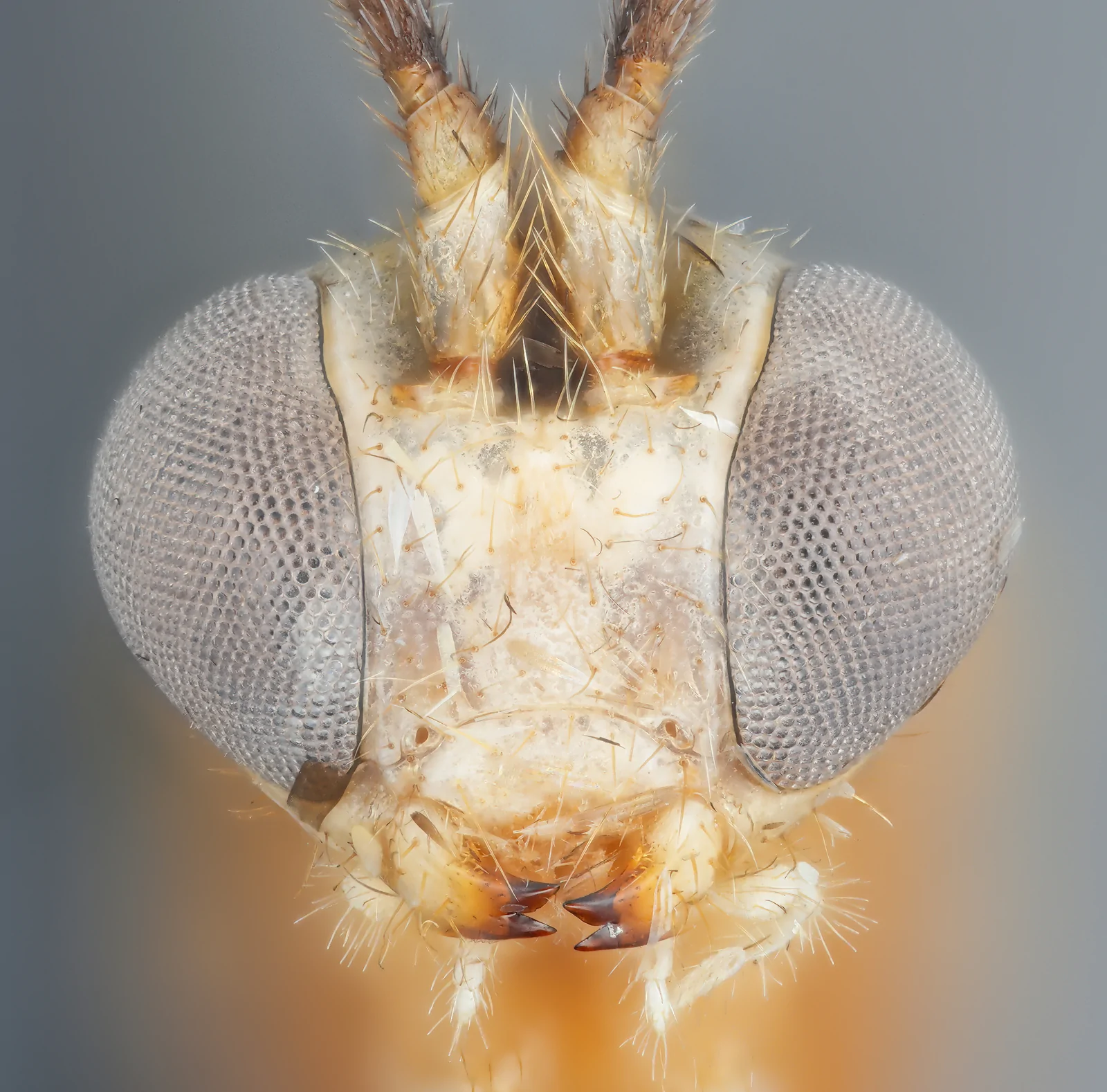
*Acropimpla lutea*, male from Uganda (ZMUT.2846), face in frontal view.

**Figure 11.**
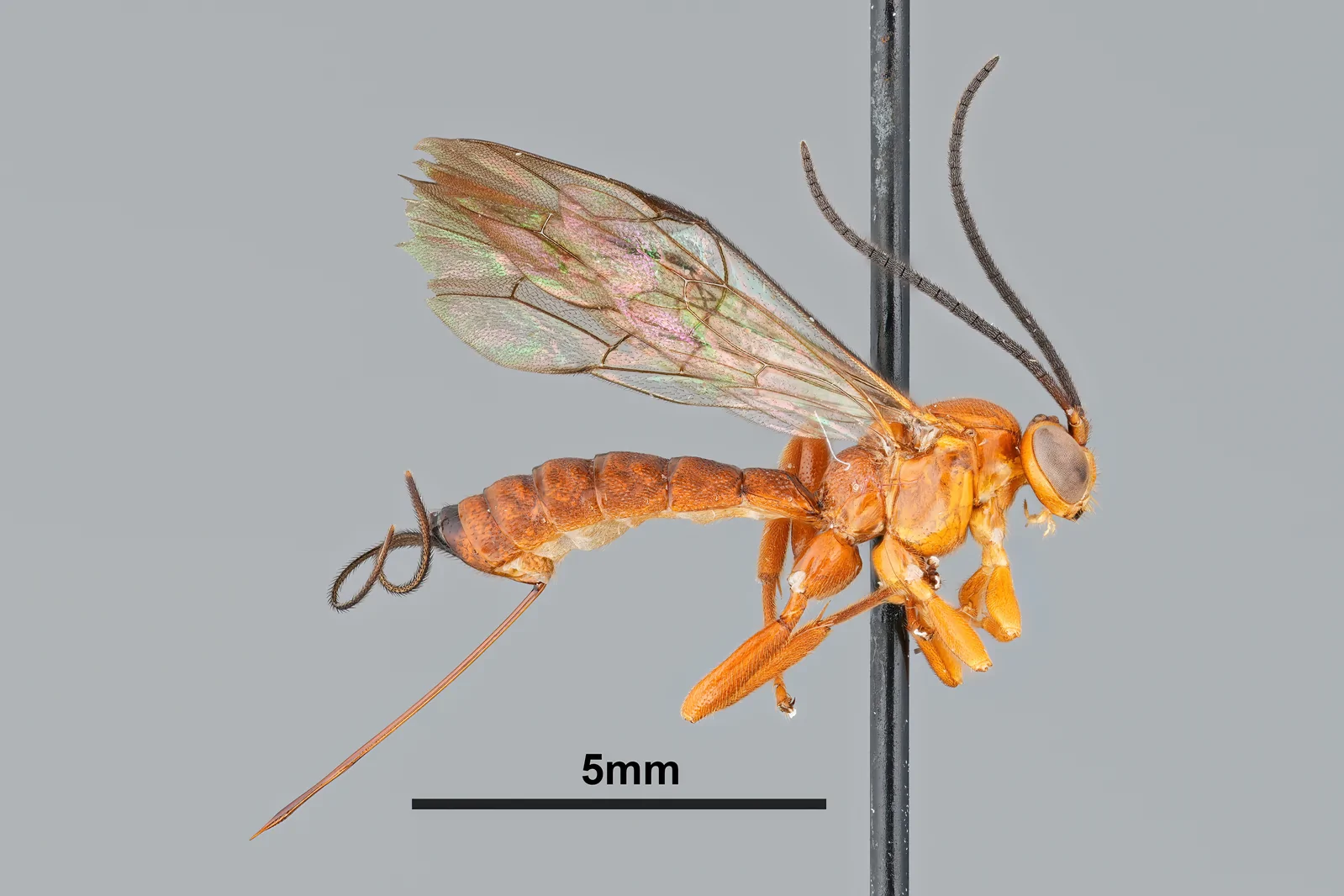
*Acropimpla lutea*, female from Uganda (ZMUT.20273), habitus in lateral view.

The male (Figs 8, 9, 10) is similar to the female (Fig. 11), except for the following: antenna with 16 flagellomeres (18–19 in females); propodeum with weak punctures (coarse in female); metasomal tergite 1 about 1.1 times as long as apically broad (0.8 in female); face whitish and head with mid-dorsal and posterior region black (face and usually also rest of head orange in females, but see notes below); metasomal tergites with extensive black areas, especially on basal and apical tergites (fully orange in female, except for occasional black bands along the apical margins). The male is also significantly smaller: fore-wing length about 3.9 mm vs. 4.8–6.7 mm in examined females.

#### Diagnosis

This species can be separated from the other known African species of the genus by any of the following: nervellus intercepted in the middle (or slightly above); mesosoma orange.

#### Distribution

Uganda (Varga 2026).

#### Ecology

In the Ugandan collecting in Kibale National Park, *A. lutea* was caught in much larger numbers in Malaise traps during the two dry seasons December–February and June–August 2015. Noticeably more were caught in disturbed forest (forest which had been selectively logged 46 years before the collecting) than in the other forest types, with none caught outside the forest in two farmland traps; it is, however, unclear if these differences between habitat types are significant.

#### Biology

No host records are known.

#### Notes

As for *A. alboscutellaris*, some females have variously developed black bands along the apical margins of tergites 2–4. Most of the smaller females have the mid-dorsal and posterior region of the head black (like the male), which are orange in the largest females with, at most, a tinge of black. Being darker is quite common in small specimens in Pimplinae species.

## Discussion

It is worth highlighting that the first male records of the known African mainland species of *Acropimpla* (*A. alboscutellaris* and *A. lutea*) presented here were collected with extensive site-specific sampling. This provides a reasonable chance of collecting both sexes of a species from the same place, making it easier to recognise the individuals of the previously unknown sex (usually males) as being conspecific with those of the known sex. Connecting the sexes of species is particularly problematic for those with a high level of sexual dimorphism, such as in some Cryptinae and Ichneumoninae genera, but Pimplinae species generally have low sexual dimorphism, as is also evident here.

Although the mainland species were collected in Uganda with an extensive sampling effort of about 382 trap months, only one male individual of each species was collected. For *A. alboscutellaris*, this may only mean that the species is uncommon in Kibale (where also only one female was collected), whereas the sex ratio of *A. lutea* in the samples is strongly skewed (54 females to 1 male, plus one whose sex has not been determined), for which the most likely explanation is male swarming behaviour (see discussion in Österman et al. (2026d)).

With respect to ichneumonids, western Africa appears to be much less sampled than central, eastern or southern Africa. The discovery of *A. alboscutellaris* from Côte d'Ivoire, being the first record of the genus from West Africa, suggests that many more taxa currently without records in the region can be common there. Ongoing sorting of our material from Côte d'Ivoire indicates that it contains further genera of Pimplinae and other subfamilies which are new records for western Africa.

The fact that the sampling in Uganda and Côte d'Ivoire collected both known mainland species, but no undescribed species (although *A. lutea* was described very recently; Varga (2026)) indicates that the species richness of the genus is not concentrated in Africa, but appears to be concentrated in the Oriental Region (see, for example, Gupta and Tikar (1976), Varga and Reshchikov (2015)).

## Supplementary Material

XML Treatment for Acropimpla

XML Treatment for Acropimpla alboscutellaris

XML Treatment for Acropimpla lucifugus

XML Treatment for Acropimpla lutea
